# Vulnerability characteristics and adaptation strategies of public health systems in different regions of China

**DOI:** 10.3389/fpubh.2025.1609377

**Published:** 2025-09-09

**Authors:** Yajun Teng, Yue Cao, Shuai Feng, Bin Han, Qinfen Gao, Zengzheng Li

**Affiliations:** ^1^Department of Emergency, The First People's Hospital of Yunnan Province, The Affiliated Hospital of Kunming University of Science and Technology, Kunming, China; ^2^Department of Emergency, Yunnan Provincial Hospital of Traditional Chinese Medicine, The First Affiliated Hospital of Yunnan University of Traditional Chinese Medicine, Kunming, China; ^3^Department of Hematology, The First People’s Hospital of Yunnan Province, Affiliated Hospital of Kunming University of Science and Technology, Kunming, China; ^4^Yunnan Province Clinical Research Center for Hematologic Disease, The First People’s Hospital of Yunnan Province, Kunming, China; ^5^Yunnan Provincial Clinical Medical Center for Blood Diseases and Thrombosis Prevention and Treatment, The First People’s Hospital of Yunnan Province, Kunming, China; ^6^Yunnan Atherosclerosis Cooperation Base of Chinese and Western Medicine, The First People’s Hospital of Yunnan Province, Kunming, China

**Keywords:** vulnerability of public health system, disease distribution, adaptation strategy, China, environment

## Abstract

Global climate change, urbanization, and environmental pollution have significantly altered the ecosystem and socio-economic structure, while also promoting a new disease risk pattern that has markedly affected human physical and mental health, increasing the complexity and uncertainty of disease control and prevention. This review aims to summarize the correlation between unique public health vulnerabilities and disease distribution in different regions of China by investigating the national and regional personalized response strategies and research progress.

## Introduction

China is located in East Asia, with a vast territory, a complex geographical environment, diverse climate types, and significant altitude differences. It is not only challenging to immediately restore equilibrium when the complex ecosystem composition is altered by extreme weather, air pollution, globalization, and other correlated factors, but it also has major repercussions, particularly the dynamic evolution of the illness spectrum. The typical ecosystem vulnerability characteristics include three dimensions: sensitivity, exposure, and adaptability. An imbalance in any of these dimensions negatively affects the system’s vulnerability. China has unique topography and landforms (the frozen Qinghai-Tibet Plateau, the towering Himalayas, the arid Tarim Basin, and the fertile Northeast Plain of China) and water distribution (the Yangtze and the Yellow Rivers), which establishes the foundation for disease distribution (1–4). Furthermore, climate differences and changes substantially affect the temporal and spatial scope of vector-borne diseases (5). Moreover, the industrialization and urbanization of emerging economies have exacerbated pollution problems (6), reduced the burden of some diseases, and promoted the prevalence of chronic diseases and environmental health risks. Comprehensive evaluation of globalization, particularly its association with the Southwestern border and Southeast Asia, as well as coastal ports such as Guangdong, China, has been under constant pressure due to the emergence of infectious diseases from other countries and cross-regional transmission. It has also increased the risk of infectious disease outbreaks (7–9). Although technological progress is crucial for disease prevention and control, it has several challenges, such as drug resistance and biosafety (10, 11). The significant changes in China’s ecosystem and socioeconomic structure have profoundly influenced the spread and distribution of diseases, forming a unique regional health risk pattern, indirectly making health issues particularly prominent.

Several epidemiological studies have shown that the causes of diseases are no longer simple and are often closely related to complex extreme weather events (such as heavy rain, high temperature, floods, cyclones, hurricanes, etc.). This can increase the incidence and mortality rates of acute and chronic diseases, as well as accelerate the incidence of severe mental health conditions (12, 13). In recent years, air pollution has caused > 1 million deaths in China each year (14), and some infectious diseases have re-emerged or even increased in certain regions (15). For instance, SARS-CoV-2 was first discovered in Wuhan in late 2019, then spread rapidly throughout China and worldwide (16). The Chikungunya virus was first discovered in Africa in 1952, and since then has caused unexpected large-scale outbreaks in Africa, Asia, Europe, and the Americas, becoming a major global health issue. On July 8, 2025, a local outbreak of Chikungunya fever was monitored in Shunde District, Foshan City, Guangdong Province, caused by an imported case.^1^ As of 24:00 on July 26, 2025, Guangdong Province reported 2,940 local cases of Chikungunya fever.^2^ Extreme weather events not only affect China, but also other developed countries like the United States. In 2020, the United States experienced 22 such events, with annual losses reaching 1 billion US dollars (17). The United Nations report (18) states that global climate change is currently the most urgent common concern. In addition to becoming the subject of international public health concern, disease and health issues are also a significant factor in China’s rapid development. The “Healthy China 2030 Strategy” guides China’s health care development, aiming to comprehensively improve the health level of the entire population and promote the coordinated development of health, economy, and society (19). To create a more sustainable and resilient society, address the challenges posed by climate change, and understand the ambitious goal of universal and global health, it is imperative to fully integrate various resources, overcome governance bottlenecks, and develop individualized and reasonable long-term strategic decisions and response measures by comprehensively understanding the characteristics of climate vulnerability and disease distribution in recent years.

There are significant regional differences in the many component characteristics associated with extreme climate events like droughts, wildfires, and floods, including the primary categories of catastrophes and the financial losses and mortality costs (20). Geospatial analysis indicates that certain regions are particularly prone to specific types of disasters. Factors such as terrain, local climate, and population density have a significant impact on the location where disasters occur (21). China is now one of the countries that are most frequently hit by disasters because of its varied temperature zones, complicated geographic setting, significant urban–rural divides, and delicate ecosystems (22). Therefore, the seven major regions of China (Northeast Region, North China Region, Central China Region, East China Region, South China Region, Southwest Region, and Northwest Region) may experience different issues related to ecosystem vulnerability, indicating health problems and economic losses. This study summarizes the representative cases of systemic vulnerability in public health issues in the seven central regions of China, as well as the common problems that exist in some areas of the country, to provide practical and feasible suggestions and measures. It also summarizes some strategic measures for each region and highlights China’s experience through particular implementation cases. By lowering exposure risk factors at their source, the goal is to control the incidence of illnesses successfully. The findings can provide a guideline to prevent and control systemic vulnerability diseases worldwide.

### Search strategy and selection criteria

For this review, PubMed was employed to search English articles published between 2000 and 2025. The search terms included combinations of “China” or “Northeast region” or “North China region” or “Central China region” or “East China region” or “South China region” or “Southwest region” or “Northwest region,” “system vulnerability” or “natural disasters” or “globalization” or “air pollution” or “ecosystem” or “extreme weather events,” “infectious diseases” or “chronic diseases” or “mental disorders” or “health issues,” “response strategies” or “strategic measures.” Furthermore, websites from different regions in China were also searched using Xinhuanet. Moreover, the official websites of various regions were investigated for data on “extreme weather events,” “natural disasters,” “infectious diseases,” and “response strategies.”

## Vulnerability of China’s regional system

**Figure 1:**
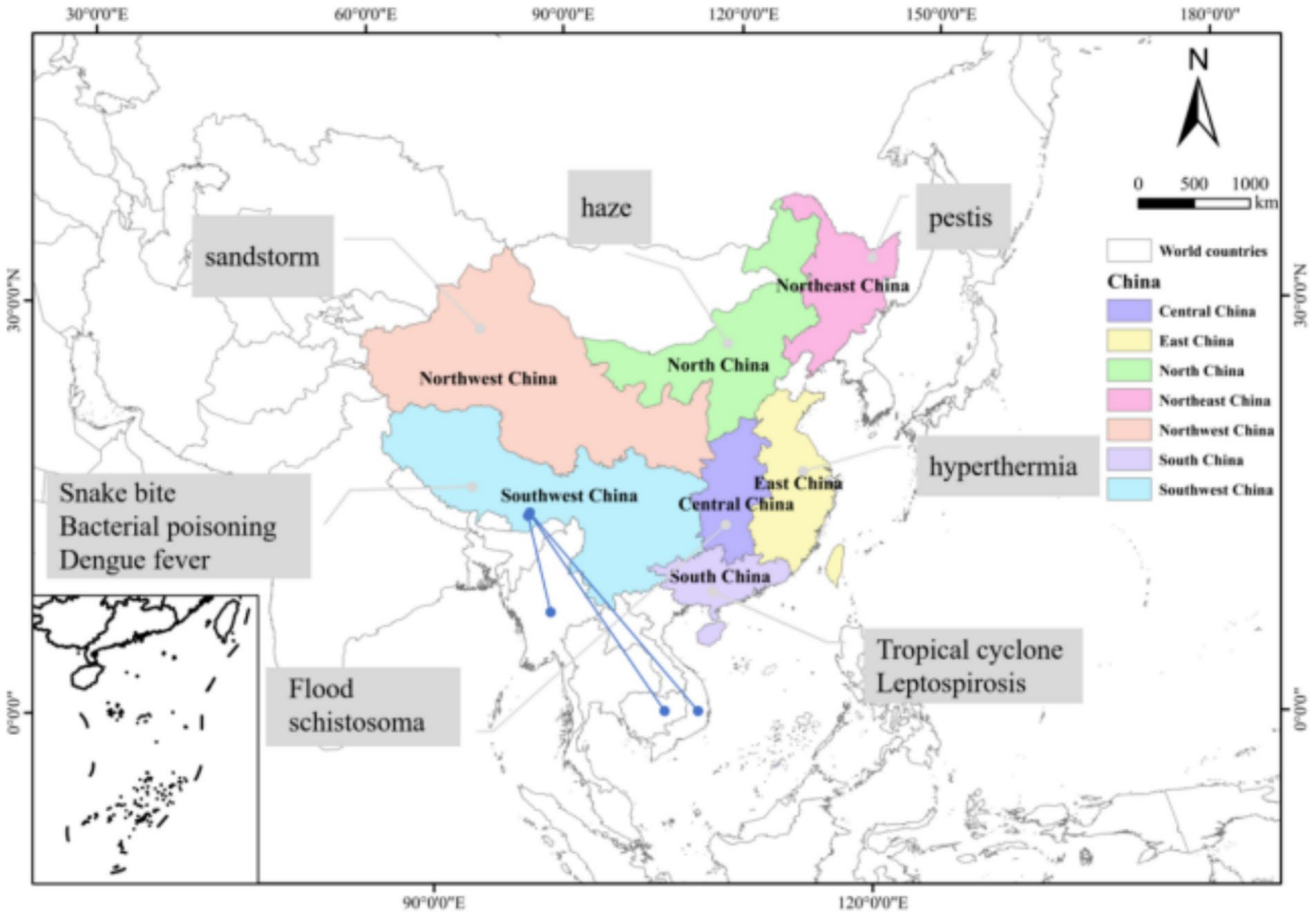
The seven geographical regions of China are represented by different colors. Each region’s representative system vulnerability and disease conditions are marked, respectively (It indicates the interaction of animal-borne diseases between southwest China and neighboring countries).

China is separated into seven geographical regions based on its different characteristics, resource distribution, and economic development, including the regions of Northeast,‌ North,‌ Central,‌ East,‌ South,‌ Southwest, and Northwest China. Diseases in different regions have different characteristics. The vertical distribution ranges from the plain areas below 500 meters above sea level to the Qinghai-Tibet Plateau with an average altitude of over 4,000 meters, spanning the Tarim Basin, Junggar Basin, Qaidam Basin, and Sichuan Basin. The distinctive regional diseases with Chinese characteristics are determined by the karst, Danxia, Yardang, and volcanic landforms found there (Figure 1).

1. Northeast China (118°-135°05′E, 38°-53°33′N): including Heilongjiang, Jilin, Liaoning, and eastern Inner Mongolia, with mainly plain terrain and a cold climate, is China’s substantial agricultural and heavy industry base. The local cold climate and agrarian production mode determine the distribution characteristics of the disease in this area

In Northeast China, greenhouse cultivation, indoor fuel combustion, and outdoor straw burning are the main production and living methods (23). The resultant environmentally persistent free radicals (EPFRs) (24) are considered the major risk factors for respiratory diseases, such as chronic obstructive pulmonary disease (COPD). A recent study revealed that COPD has a relatively high prevalence in Northeast China on a national scale (25). Furthermore, solid fuel combustion increased the risk of lung cancer (26). The primary strategies for preventing disease depend on the disease’s exposure factors. Residents’ lifestyles are altered and their living conditions are improved by pertinent public health laws and policies, such as the use of biogas in place of coal and the burning of straw (27). The government places a strong emphasis on addressing the health issues caused by COPD. China has made it a goal to lower the death rate associated with COPD and other chronic respiratory diseases among individuals under 70 to 8.1/100,000 or fewer by 2030. The “Implementation Plan for the Prevention and Control of Chronic Respiratory Diseases” was released concurrently by the relevant departments, which calls for actively reducing the COPD risk factors, enhancing the prevention and control system, standardizing the diagnosis and treatment procedure, and improving support for comprehensive guarantees and scientific research. Coal use has declined, car emissions have been successfully managed, and nitrogen deposition in Northeast China has significantly decreased thanks to the tireless efforts of the government and everyone else, as well as because of the change in agricultural methods (28).

Inner Mongolia is home to a world-renowned dairy cluster and a well-developed livestock industry. It is the nation’s top producer of wool and mutton. Although the industry sector is expanding, it has promoted certain disease-related issues. Human brucellosis is most common in Inner Mongolia and the provinces or autonomous regions that surround it (29, 30). Large-scale sheep farming, frequent animal trading, cross-regional circulation of dairy products, and a weak grassroots epidemic prevention system all increase the risk of brucellosis transmission and resurgence. Strengthening personal protection, early detection, and early treatment can effectively control the trend of brucellosis (31).

Plague is a zoonotic disease that spreads through fleas and is transmitted by rodents carrying the pathogen (32). Historically, it has caused three major pandemics, resulting in over 200 million deaths (33). There are indications that the prevalence of plague is increasing in the Americas, Asia, and Africa as a result of climate change (34). The Siberian marmot (*Marmota sibirica*) is widely distributed in the grasslands of Northeast China (35), whereas the *Marmota himalayana* is widely distributed in the Qinghai-Tibet Plateau (QTP) (33); these two together form the largest natural infection area in China. The literature has indicated that plague spreads faster in areas with low population density and a high proportion of grassland or forest (36). Furthermore, the warming trend in the QTP region (37) and the developed tourism industry (38) further increase the risk of plague transmission. Some studies have indicated that an increase of 1 °C in spring temperature elevates the risk of pathogen infection in gerbils by 59% (39). Further, heavy rainfall also increases the risk of plague transmission (40). Various fleas can survive in the Alxa Desert in Inner Mongolia, China, which is a typical arid region of Asia with a hot, dry climate. Fleas are the primary carriers of various zoonotic diseases and frequently parasitize rodents (22, 41). Therefore, novel strategies are required for the prevention and control of diseases like plague.

2 North China (110.22°-119.85°E, 34.58°-42.37°N): covers Beijing, Tianjin, Hebei, Shanxi, and central Inner Mongolia, has diverse terrain and a temperate monsoon climate, and is the political and cultural center of China

In China, non-communicable illnesses have progressively become the major cause of early mortality and disability (41), with air pollution listed as one of the four major risk factors. In recent years, the environment, human health, and economy have all suffered due to deteriorating dust weather in northern China. The number of cross-border sandstorms from Mongolia to China has been increasing from 1987 to 2022 (42). The concentration of PM10 (particles with a diameter of less than 10 μm) in Beijing increased to over 7,000 μg M-3 on March 15, 2021, due to a super sandstorm (43). Furthermore, the spring of 2023 had the highest frequency of spring dust weather in the previous 10 years due to a protracted sandstorm outbreak caused by the cold front of the Mongolian cyclone and the dust source area (44). Sandstorms have oxidation potential (OP) and EPFR, inducing oxidative stress. They are closely related to the incidence of circulatory system disorders (45–47) and respiratory system diseases (48). Moreover, the incidence of hospitalization among patients was significantly correlated with exposure to sandstorms (49). Moreover, recent studies have discovered that exposure to PM2.5 directly induces nephrotoxicity, which is linked to five different forms of kidney disorders (50). A meta-analysis indicated that PM2.5 exposure is significantly associated with liver dysfunction, chronic liver diseases, liver cancer, and colorectal cancer (51), as well as an increase in the incidence of lung cancer (52). In addition to threatening the environment and public health in northern China, sandstorms are progressively spreading to the center and southern parts of the country, endangering the environment and public health worldwide, and 150 nations have been directly impacted thus far (53). Despite the initiatives proposed by the United Nations, it is still influenced by various factors. Therefore, international cooperation and multidisciplinary health research are still needed.

As China’s economy expanded quickly, a significant increase was observed in air pollution between 2012 and 2017. The green finance index of Liaoning experienced extreme smog pollution, and the Beijing-Tianjin-Hebei region exhibited an overall negative trend between 2010 and 2017 (54). Smog not only has a significant negative impact on the economy and society, but also poses a considerable threat to people’s health.

Urban greening is a valuable strategy that is being employed to reduce the premature mortality rate caused by non-communicable diseases by one-third by 2030. Green spaces can alleviate environmental pollution, restore energy consumption of the body, and help people build health capabilities (55). A systematic study revealed a positive association between green spaces and mental health, cardiovascular outcomes, and overall health (56).

3 Central China (108°21′-116°39′E, 24°38′-36°22′N): includes Henan, Hubei, and Hunan provinces, has plain and hilly terrain, developed water systems, and is an important agricultural and industrial base in China

Floods have become more frequent and longer in duration due to global warming, which has had a negative influence on China’s social and economic structure as well as the health of the environment. The most catastrophic disaster since 1998 occurred in 2016, when the Yangtze River Basin experienced a devastating flood brought on by the El Niño phenomenon.

Hunan Province in China is regarded as one of the provinces most severely affected by floods in recent years (57). Water-borne illnesses include bacillary dysentery, cholera, and typhoid/paratyphoid. The risk of disease transmission rises when floodwaters contaminate drinking water (58). A study found that after the flash flood and waterlogging in Changsha City, the incidence of typhoid/paratyphoid and bacillary dysentery, which are related to intestinal infections, increased. The higher the level of storm floods, the higher the risk of disease outbreaks (59).

The saying “The danger of the Yangtze River lies in Jingjiang” refers to the distinctive geographic setting of the Jingjiang stretch of the Yangtze River in Hubei Province, which has historically been vulnerable to recurrent floods. In 2016, 20.8 million people in Hubei Province were impacted by the flood (60). Floods not only cause direct losses to people and facilities but also increase the risk of new infectious diseases. Hemorrhagic fever with renal syndrome (HFRS) is a zoonotic disease mainly transmitted by rodents carrying the Hantavirus (61). Previous literature suggests a positive correlation between hydrological conditions and the incidence of HFRS (62). According to some studies, HFRS will be a major threat to mainland China during the 21st century, particularly in the country’s northeast, east, and center. Furthermore, since the rodent population density may increase after floods, the risk of HFRS in low-altitude and flat terrain locations close to rivers should be assessed, and short-term HFRS preventative strategies should be formulated (60).

Schistosomiasis is a disease transmitted through the intermediate host, the Oncomelania snail (63). Although it has been successfully managed through extensive preventative and control methods, the central China region (Dongting Lake, Poyang Lake) has found it extremely challenging to prevent and control schistosomiasis due to the regular occurrence of floods (64). Comprehensive transformation of agriculture, water conservancy, forestry, health, and the environment is beneficial for the prevention and control of schistosomiasis (65). Of them, controlling the water levels at the Three Gorges Dam (TGD) to limit the population of Oncomelania snails has been considered a successful and effective case, as it has considerably decreased the snails’ density, infection rate, and potential for human interaction with (66).

4 East China (113.55°-122.87°E, 21.9°-38.32°N): It covers Shandong, Jiangsu, Anhui, Shanghai, Zhejiang, Jiangxi, Fujian, and Taiwan. The terrain is mainly hilly, with basins and plains. The climate is diverse, and the economy is developed

The increase in global warming and carbon emissions directly increases the surface temperature. High temperatures can lead to heatstroke and increase the risk of circulatory and respiratory system failure (67). The COVID-19 pandemic and hot weather are no coincidence, as unprecedented heatwaves increase the number of infectious diseases (68, 69). The urban heat island effect may be more pronounced in the more economically developed eastern coastal districts, potentially intensifying and prolonging heatwaves (70, 71). It has been estimated that nationally, the attributable fraction of non-accidental deaths due to high temperatures will increase from 1.9% in the 2010s to 5.5% in the 2090s, with an estimated attributable fraction of 6.3% for cardiovascular system deaths and 7.7% for respiratory system deaths (72). The literature suggests that the annual heat stress index (HSI) in China has generally risen, and by 2,100, will increase by 7.96 °C based on the SSP5-8.5 scenario (73). Furthermore, heatwaves have been found to significantly increase the risk of death among the older adults suffering from Alzheimer’s disease and other dementias (74). To prevent infectious diseases under the influence of high temperatures, government policies and individual protection awareness need to work together. The number and length of case encounters can be decreased by non-pharmaceutical intervention techniques (NPIs). According to recent studies, the effective creation of biodegradable and sustainable filter materials offers a fresh, alternative approach to air quality control (75). Moreover, strengthening drug intervention can control the epidemic and reduce the risk of transmission (76, 77). A study showed that high temperatures can increase the burden of cardiovascular diseases. Therefore, reducing emissions and implementing strategies can mitigate health risks related to human-induced climate change (78).

Poyang Lake is the largest freshwater lake in China. The microbial population in the marshes around Poyang Lake’s shore experienced substantial alterations during the severe and quick drought event in 2022. The diversity of denitrifying and DNRA bacteria declined (79). The increase in nitrous oxide emissions and nitrogen retention increased the greenhouse gas emissions and raised the risk of lake eutrophication, intensifying the positive feedback between climate change and nitrous oxide emissions (80).

5 South China region (105°29′-117°14′E, 18°10′-26°15′N): includes Guangdong, Guangxi, Hainan, Hong Kong, and Macao. The terrain is mainly composed of hills with a humid and hot climate. It is the forefront of China’s opening up to the outside world

China borders the Northwest Pacific Ocean and is situated in the eastern region of the Eurasian continent. Therefore, she is one of the nations that is most severely impacted by tropical cyclones worldwide (81). Since tropical cyclones often make landfall in Guangdong Province, the losses caused by tropical cyclones in Guangdong Province are greater than those in any other province (82). In addition to their direct effects on the environment, economy, and society, tropical cyclones can have an indirect effect on public health. Studies have indicated that acute hemorrhagic conjunctivitis, diarrheal infectious diseases, leptospirosis, and hand-foot-mouth disease are among the infectious diseases that have been linked to tropical cyclones (83–85). A study showed that tropical cyclones may increase diarrheal infectious diseases in Guangdong Province (86). Another study revealed that tropical cyclones may increase the risk of hand-foot-mouth disease in children under 6 years old (87). The danger of mosquito-borne illnesses like dengue fever rises in conjunction with the temperature fluctuations and precipitation brought on by tropical storms, which may be suitable for mosquito reproduction. Since an unprecedented dengue fever outbreak occurred in Guangdong Province in 2014 (88), from 2007 to 2017, about 94% of dengue fever cases in China occurred in Guangdong Province (89). Furthermore, it has been observed that in the Pearl River Delta, tropical cyclones are linked to a higher prevalence of primary dengue fever, with the risk peaking 5–9 days following the occurrence. The risk is greatest when lagging 5–9 days (90).

Leptospirosis is an animal-source disease (91), and the risk of leptospirosis outbreak is significantly increased by flood events (92). Leptospirosis has historically been more common in the Southwestern and Southeast regions. The disease is mainly linked with farmland and marshes in the Yangtze River Basin and its Southern areas, flood exposure in the Yellow River Basin and Northern China, and the buildup of water following heavy rains in low-lying plains (93). The transmission of leptospirosis is promoted by climatic variables such as heavy rain, floods, humidity, and high temperature (94). Studies have found that leptospirosis outbreaks can be avoided in locations that are vulnerable to extreme weather events like floods and heavy rain by implementing prevention and control measures (95).

6 Southwest Region (78°-110°E, 21°-34°N): covers Sichuan, Guizhou, Yunnan, Tibet, and Chongqing. The terrain is mainly composed of plateaus and basins, with diverse climates and rich cultures

Since the COVID-19 pandemic in 2019, animal hosts have attracted much attention, including bats, civets, pangolins, and other species (96). Zoonotic infectious illnesses have increased as a result of the varied effects of climate change on the species diversity of animal hosts. Bat species richness has increased significantly in southern Yunnan, Myanmar, and Laos as a result of climate change, and bats are currently thought to be the source or intermediate host of Middle East Respiratory Syndrome (MERS) (97), Ebola (98), SARS-CoV-1 and SARS-CoV-2 Rift Valley fever (RVF) (99), etc. At present, the coronavirus strain found in bats in southern Yunnan is the closest to SARS-CoV-1 and SARS-CoV-2 (100). Hunting, eating patterns, and agricultural practices that involve intimate contact with pathogen-carrying wild animals are all linked to the zoonotic disease outbreak (101). Therefore, it is crucial to protect natural habitats, implement strict regulations on wildlife hunting and trade, establish appropriate animal welfare standards, and adjust dietary and medicinal customs (96, 102).

Dengue fever is a mosquito-borne disease that is prevalent worldwide (103). The invasion of alien species and changes in the global environment have become major contributors to the development of dengue disease and have made its prevention and control extremely difficult (104, 105). However, China’s public health prevention and control efforts continue to prioritize preventing local dengue fever transmission; thus, environmental intervention and risk area identification are essential. It has been observed that Southeast Asia is the primary source of dengue fever invasions, and Yunnan, China, which borders Laos, Vietnam, and Myanmar, is under intense pressure to prevent the invasion and reduce dengue fever prevalence (106). Temperature affects the physiological process of the dengue virus-mosquito-host chain, thus becoming one of the important driving factors for dengue fever (107). *Aedes aegypti* and *Aedes albopictus* are the primary vectors for dengue fever transmission, which can survive at 10 °C–40 °C and spread diseases in a temperature range from 16.2 °C to 34.5 °C. Higher temperatures may increase the expected lifespan of mosquitoes, promoting a longer exposure time to dengue fever. The Southeastern part of China is affected by the East Asian monsoon, with higher summer temperatures (108); therefore, this region has a significant impact of dengue fever (106). Due to climate change, the suitable environment for vector insects is expanding (109), and the risk of dengue fever transmission is increasing.

One of the highest-altitude towns in the world, Lhasa is situated in the middle of the Tibetan Plateau and experiences significant intensities of ultraviolet (UV) radiation. The literature has indicated that UV radiation is one of the major risk factors for three pigmentary skin illnesses, which are far more common in high-altitude regions than in low-altitude ones (110). Due to climate warming and the prolonged duration of ozone, earlier seasonal snowfall and ice melting, more and more people are exposed to ultraviolet radiation, which seriously affects human health (111).

Yunnan, situated in the tropics and subtropics, boasts rich biodiversity, earning it the title of having one of the richest plant communities in the world, which in turn makes its fungal resources abundant (112). Edible mushrooms come in many different varieties and are both tasty and nourishing. The living environment of mushrooms has altered as a result of ecological changes, and both their poisonous and nutritious components have changed as well. Mushroom poisoning incidents are one of the main causes of death from foodborne diseases in China (113, 114). Due to its unique climate and topography, it is the province with the most extensive variety of snake species in the country and a high incidence area of snakebite poisoning in China. Furthermore, this region borders the frontier, with ethnic minorities and poverty-stricken areas (115).

1. 7 Northwest Region (73°-111°E, 31°-49°N): includes Shaanxi, Gansu, Qinghai, Ningxia, and Xinjiang. The terrain is mainly composed of plateaus and deserts, with an arid climate and abundant resources

Severe air pollution has been associated with sandstorms. Several studies have indicated that during sandstorms, the hospitalization and mortality rates increase significantly (116, 117). Asian sandstorms mainly originate from the arid regions in the northwest and north of China and Mongolia, and spread to East Asian countries (118). Furthermore, sandstorm events have been positively correlated with the number of visits for asthma (119), stroke (120), congestive heart failure (121), and conjunctivitis (122).

Climate warming is melting the glaciers in the Asian mountainous regions (123), and the permafrost layer on the Qinghai-Tibet Plateau. When the frozen viruses are discharged, there are more chances for the viruses to propagate throughout the habitats and migrations of wild migratory birds. When the frozen viruses are discharged, there are more chances for the viruses to propagate in the wild migratory birds’ habitats and migrations (124). In 2005, a large-scale H5N1 avian influenza epidemic broke out in the Qinghai Lake area, resulting in the death of thousands of migratory birds (125). This indicates that the Qinghai Lake area has become a significant source of H5N1 avian influenza.

1. 8 Common Systemic Vulnerable Diseases in China

Cryptosporidiosis is a zoonotic disease transmitted through the fecal-oral route (126), which can cause abdominal pain, diarrhea, and acute gastroenteritis in infected hosts (127). It is highly contagious and has become a globally highly concerning acute infectious disease (128). Research has indicated a strong correlation between climate change and Cryptosporidium’s biological adaptability, and climate change may be a major factor influencing the species’ survival, resulting in its extinction, re-distribution, resurgence, or re-emergence (129). Furthermore, regions in Northeast China (Jilin Province and Heilongjiang Province), Southwest China (Yunnan Province), and Northwest China (Xinjiang Uygur Autonomous Region) are significantly affected by climate change and may be suitable for the survival of Cryptosporidium. Future climate change may provide China with more favorable habitats for Cryptosporidium (130). The Maxent model can predict the distribution of Cryptosporidium and formulate a public health strategy covering “parasite–host-environment,” strengthening real-time monitoring, increasing information exchange and communication, improving the monitoring reporting system, and improving interdisciplinary linkage mechanisms (130).

China’s rainfall is influenced by various large-scale ocean–atmosphere patterns, including El Niño-Southern Oscillation (ENSO), Pacific Decadal Oscillation (PDO), Indian Ocean Dipole (IOD), and Atlantic Multidecadal Oscillation (AMO) (131). These patterns play an important role in the occurrence of extreme drought events (87, 132). Wildfires are becoming more frequent as a result of drought and global warming, which is extremely dangerous for human life, health, and productivity (133, 134). Carbon-containing gases and tiny particulate matter are released when wildfires burn, polluting the air and raising the risk of cancer, heart disease, and respiratory ailments for firefighters who fight fires (135). The development of new protective technologies against wildfire combustion pollutants is of vital importance.

Under the influence of multiple pollutants such as PM2.5, PM10, O3, NO2, CO, PAHs (Polycyclic Aromatic Hydrocarbons), SPM (Suspended Particulate Matter), SO2, NO, radon gas (136–139), multiple organ systems such as the cardiovascular system (45), respiratory system (48, 140), digestive system (51) nervous system (141–143), eye-related diseases (144, 145), urinary system (50, 146), and mental health (147, 148) are all affected to varying degrees. The air pollution levels in many Asian nations, particularly China and India, are far greater than those in wealthy nations. In a white paper, the Allergy, Asthma and Clinical Immunology Association of Asia-Pacific highlights the current state of air pollution in the Asia-Pacific area and urges national and international health and environmental organizations to take action (149).

## The connection between China and international characteristic systemic vulnerability diseases

The total length of China’s land borders is approximately 22,000 km, making it one of the countries with the longest land borders in the world, bordering 14 countries. The “Belt and Road Initiative” has had a tremendous impact on trade openness and tourism expansion in recent years, which has greatly boosted people’s mobility both domestically and globally. Moreover, this has raised the possibility of infectious illness transmission. China is not the only country experiencing climate change. Some ecosystem-related diseases are determined by the unique geographical location. Therefore, there is a certain correlation among global system-related diseases in different countries.

Food security is severely compromised by Schistocerca gregaria, which has caused famine, particularly in South Asia, the Middle East, and Africa, important regions of the world. The biological behavior of locusts is strongly influenced by hydrological and climatic factors, with temperature, wind direction, and rainfall all having distinct effects (150). Studies have shown that a warmer climate promotes locust outbreaks, and new hotspots will emerge in central and western Asia. Early cross-regional cooperation is helpful for global coordinated control of the food security threat brought by locusts (151). It has been identified that the El Niño (Southern) oscillation (ENSO) is the primary climate oscillation in southern China. ENSO leads to a higher incidence of crop pests. EnSO-driven cyclic patterns identify the pests’ source locations, which makes it easier for them to migrate to China. Early cross-regional collaboration is beneficial for the global coordinated management of pest risks to food security (152).

## Measures for responding to vulnerable diseases in the Chinese system

### Ecological vulnerability and adaptation measures

**Figure 2:**
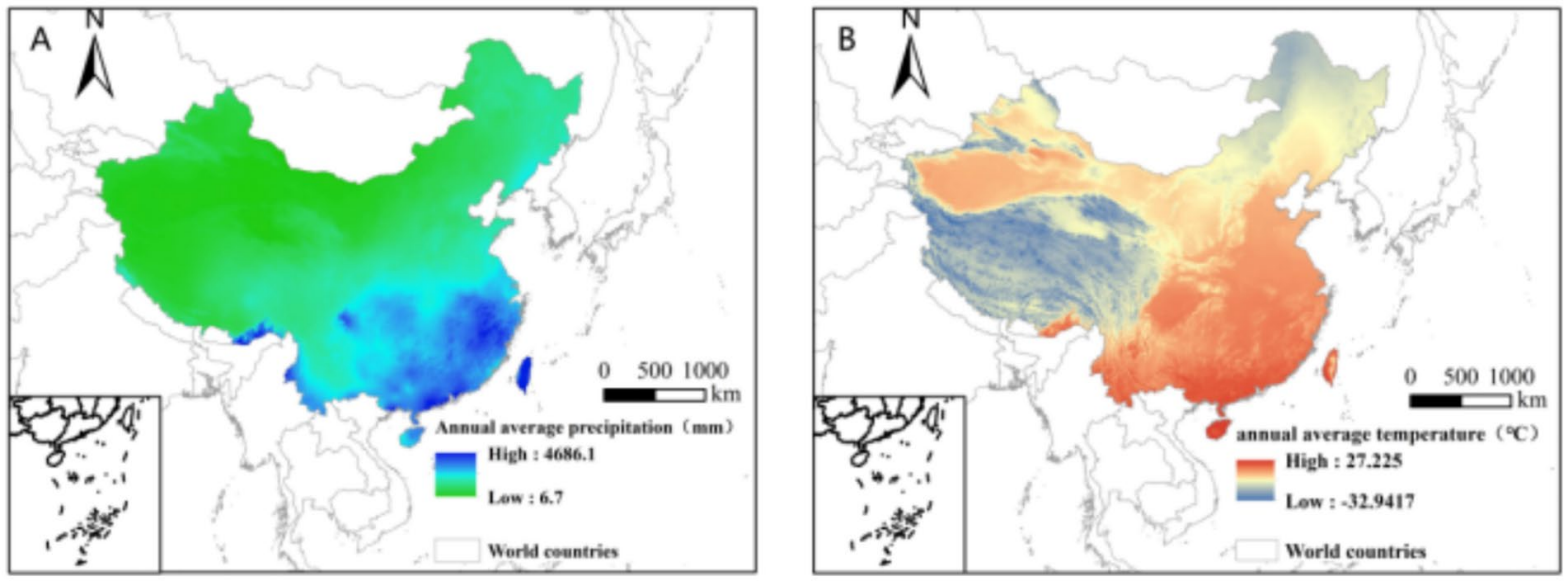
Annual average precipitation (mm) **(A)** and temperature (°C) **(B)** in China in 2023.

The primary and most obvious effects of climate change in China over the last three years have been an increase in yearly precipitation and a temperature rise (Figure 2). The incidence of natural disasters has increased to different degrees in each region (Table 1). Certain areas have put in place specific emergency response procedures and precautions for various extreme weather occurrences (Table 2).

**Table 1:** The manifestations of system vulnerability in some regions of China over the past 3 years due to climate change.

Regions	Natural disaster events	Coping strategies	References
Northeast China 118°-135°05′E, 38°-53°33′N	In 2024, the precipitation in Liaoning Province reached 883 mm, setting a new historical record since 1951; In 2023, in the southwestern part of Jilin Province, due to Typhoon Dujuan, the precipitation exceeded the historical record; In November 2023, rare heavy snow occurred in many places, and the temperatures in many areas dropped below −40 °C; In 2023, the average temperature in Jilin Province was 5.8 °C, which was 1.1 °C higher than the previous year; In 2024, the average temperature in Liaoning Province was 10.3 °C, which was 1.3 °C higher than the previous year.	Upgrading of urban and farmland drainage systems (case of full-load drainage of irrigation canals in Shenyang City in 2024) Reinforcing windproof and frost-resistant facilities (promoting the design of wind-resistant and snow-proof greenhouses) Establishing a full-chain emergency mechanism covering “warning release – disaster response – resource allocation – post-disaster recovery”	https://news.qq.com/rain/a/20250321A08LWS00?web_channel=wap&openApp=false&suid=&media_id= https://m.yunnan.cn/system/2024/01/11/032905980.shtml https://finance.sina.com.cn/jjxw/2023-11-06/doc-imztrmty3805686.shtml?cref=cj
North China 110.22°-119.85°E, 34.58°-42.37°N	In the summer of 2023, the Huang-Huai region experienced the strongest heatwave since 1961, surpassing the historical extreme value.		(186)
Central China 108°21′-116°39′E, 24°38′-36°22′N	In December 2023, a severe cold wave hit the Central China region, causing snowfall in many areas to exceed the historical extreme values for the same period.		https://www.mem.gov.cn/xw/yjglbgzdt/202401/t20240120_475697.shtml
Eastern China 113.55°-122.87°E, 21.9°-38.32°N	In the summer of 2023, temperatures in Zhejiang, Fujian, and other places exceeded 40 °C; The transition of extreme weather is accelerating, with hot spells rapidly turning into heavy rains, increasing the difficulty of disaster prevention. The National Marine Environmental Forecasting Center predicts that the East China Sea coast may face 4–6 disastrous typhoons and cold waves in 2025.		https://finance.sina.com.cn/jjxw/2023-12-29/doc-imzzsuqv5663290.shtml?cref=cj https://m.sohu.com/a/853547265_121119270/?pvid=000115_3w_a https://news.qq.com/rain/a/20250328A098MZ00?web_channel=wap&openApp=false&suid=&media_id=

**Table 2:** The manifestations of some characteristic system vulnerabilities in China and the corresponding countermeasures.

Exposure events	Strategies	Representative	References
Air contamination	Coal-to-electricity transformation measures The strategy of coordinated development of the Beijing-Tianjin-Hebei region Forest ecological restoration project (FERP) Judicial innovation for enhancing the forest carbon sink Zero-waste city (ZWC) plan Green finance (GF) initiative Global public health cooperation under the BRI (One Belt, One Road) initiative Green City	“2 + 26” Provincial-level Urban Power Dispatch Model; Recommendations for Implementing the “Three Transformations and One Integration” in Thermal Power Plants under the Background of “Carbon Peak and Carbon Neutrality”;	(177–182, 187)
Flood disaster	Urban Flooding Image Recognition and Early Warning System Establishing an elastic urban drainage system through a comprehensive flood risk index 3. Regional Joint Governance	Utilize the original facilities of TensorFlow to implement the display of warning information on the LED screen, video surveillance, and sound column alarm.	(171, 183, 188)
Drought	Research and development of drought-resistant crops Monitoring of crops and drought climate prediction, ensuring food security		(189, 190)

### Strategies for addressing the gaps in the health system and infrastructure

For the outbreak of infectious diseases, not only does the health system need to respond actively, but also infrastructure construction needs to respond promptly. China has its tailored response strategies for communication with the world (Table 3). However, China currently lacks extensive practical experience in coping with several infectious diseases around the world, and it must continue to learn from and draw on other nations’ experiences (Table 4). From the standpoint of public health policies, no nation can effectively address the issue of global climate change. Thus, international cooperation is crucial (153).

**Table 3:** China’s countermeasures for diseases related to system vulnerabilities.

Disease threat	China	References
Dengue	Early warning system, interdisciplinary cooperation, environmental and ecological intervention measures, scientific research, and the adoption of the “Health One” approach	(166)
Schistosomiasis	Design of the South-to-North Water Diversion Project for Control of Schistosomiasis: River hardening, facilities for trapping and blocking schistosomes, and sedimentation ponds; Regional strategies such as the “Elimination of Schistosomiasis in Hunan Province (2016–2025)” plan; The implementation of the “Snail Elimination – Water Improvement – Health Education” tripartite strategy in endemic areas.	https://www.gov.cn/jrzg/2007-08/08/content_709291.htm
Variant virus	Vaccine research and development in the Guangdong-Hong Kong-Macao Greater Bay Area, specifically in Shenzhen and Zhuhai, are laying out mRNA vaccine production bases.	(191)
Helopyra	Carry out cross-border malaria monitoring in cooperation with other countries (such as the “Lancang-Mekong Malaria Control Project” in Yunnan Province)	
Influenza	Establishment of an early warning system for airborne infectious diseases Significant nonlinear and delayed associations between ambient temperature and the incidence of influenza	(192)

**Table 4:** Countermeasures in other areas for diseases related to system vulnerabilities.

Disease threat	Other areas	References
Monkeypox	On September 20, 2022, a total of 19,827 confirmed cases were reported by 29 European Union countries. Countermeasures: Vaccine genetic engineering	(184, 193)
Cholera	Since 2023, large-scale cholera outbreaks have continued to occur in the armed forces of Sudan. Countermeasures: The single-dose kOCV vaccination campaign is combined with the WASH (Water, Sanitation, and Hygiene) program.	(194–196)
H5N1 and other avian influenza strains	As of January 13, 2025, the Centers for Disease Control and Prevention (CDC) of the United States reported 1,410 cases; Countermeasures: Update the “Global Influenza Control Framework,” multi-sectoral collaboration, and research and reserve antiviral drugs.	(197–199)
Pest management	Environmental DNA (eDNA) has emerged as a potent tool for the rapid and precise identification of individual organisms and species assemblages across various matrices, including air and soil.	(200)

### Regional adaptability ecosystem prediction model

Significant temporal and spatial disparities, severe wealth disparity, and differing levels of resource usage all make it harder to achieve unified governance and increase the ecosystem’s susceptibility in China. There are significant disparities in economic status and urbanization development between the eastern and western regions. Thus, they may face different health issues and ecosystem vulnerabilities, and there are also significant differences in the emergency response capabilities of the health systems. Based on the modified gravity model social network analysis, a study evaluated the network structure characteristics of China’s urban ecological resilience (UER) and revealed that the economically developed areas along the eastern coast had a significant “radiation effect” in spatial correlation. Furthermore, they indicated the importance of strengthening coordination and cooperation among regions to enhance UER (154). Extreme disaster events occur differently in coastal and plain areas due to significant climate changes, and each region has different priorities for emergency response. A study evaluated the patterns of flood resilience over 3 years using ArcGIS software to dynamically track each city’s level of resilience. The flood resilience level in the Pearl River Delta was very different, with the levels in the central and southern cities being higher than those in the eastern and western regions. Moreover, an innovative method was constructed to comprehensively assess the flood resilience of cities, which is suitable for quickly and accurately evaluating the short-term spatial and temporal evolution trends of urban flood resilience (155). Further research is needed to confirm that widespread adoption of this approach will effectively assess the temporal and spatial evolution patterns of floods in each region, improving flood prevention capacities, reducing health risks, and minimizing economic losses.

#### Southwest region

As an ecologically barrier and an underdeveloped area in the southwest, Yunnan’s social and economic development mainly relies on agriculture. Therefore, its ecosystem is susceptible to climate change (such as droughts and floods). However, Yunnan’s climate zones are complicated, and its altitude range is broad, allowing for exact classification of various places based on sensitivity levels. The ecosystem’s high sensitivity could have more negative effects. The ecosystem’s risk characteristics must be thoroughly evaluated, and appropriate action must be taken to guarantee the sustainable development strategy’s seamless implementation. A research group constructed a “climate-livelihood” framework by theoretical integration (IPCC + sustainable livelihood + human-environment coupling) and indicators localization, to build a vulnerability assessment system applicable to rural areas in Yunnan (156). This framework’s limitations include the absence of policy frameworks and the challenge of gathering subjective data. However, it takes into account the comprehensive variables of ecology, livelihood, and climate, and hence is appropriate to most rural locations (such as the southwestern mountainous regions).

#### Central China region

Hunan Province has a subtropical monsoon climate and is situated in China’s Yangtze River Basin. This region experiences flooding often (on average, 1 to 3 times a year between 2004 and 2011), which is followed by a significant rise in infectious diarrhea cases. This also seriously threatens the lives, health, and property safety of the people. A study found that focusing on precise intervention (water source guarantee, sanitation facilities), phased response (during and after the flood for 2 weeks), and resource allocation (in economically backward areas) can reduce the risk of diarrhea outbreaks after floods (157). Although the above research results considers the disease-related risk factors, this approach can still be employed in most southern regions. Furthermore, the flood resilience assessment system of Lagos Metropolis can be borrowed to provide a scientific basis for emergency response before and after major floods in various cities (158). Some researchers have modeled and conducted dynamic analysis of flood events in China and the UK. More real-world experiments and the development of flood-health risk warning systems might be required in the future to modify the preventative and control tactics on the fly (159).

#### East China region

Shanghai is one of the representative areas of economically developed China. Due to urbanization, the population’s morbidity has been affected in multiple ways by the extremely hot and muggy weather resulting from the combination of high temperatures and humidity. Studies have used the wet-bulb globe temperature (WBGT) to assess health risks, providing an operational basis for climate adaptation strategies (such as warning systems, urban planning) (160). For areas with severe, extremely hot, and humid weather (Chongqing, Guangdong), the WBGT can be included in the early warning system of public health, and the urban heat island effect can be alleviated through green space planning and building ventilation optimization to adapt to the inevitable climate warming trend in the future. However, further scientific investigation is required to validate this finding.

### China’s strategy and cross-regional cooperation strategy

To address the problems caused by the vulnerability of China’s and the global public health systems, professionals across China are actively seeking, innovating, and optimizing various prediction models to formulate strategies that are highly operational and beneficial. Currently, initial results have been acquired from areas affected by the urban heat island effect, focusing on dengue fever prevention and control, as well as flood prevention after heavy rain. Other areas of China and other nations can refer to and learn from the cross-regional cooperation cases. However, in the face of the unpredictability of the future, there is still much room for improvement.

#### Blue-green spaces (BGS)

Extreme weather events, such as droughts, high temperatures, and floods, are increasingly common due to the urban heat island effect and global warming, significantly impacting the lives and health of local communities. Studies have shown that blue-green spaces (BGS) and urban green spaces (UGS) can mitigate the adverse effects of extreme climates on the urban environment. Research in Changchun and Ningbo of China indicates that UGS have a strong cooling effect on the surrounding environment, which is concurrent with the increase in Land Surface Temperature (LST) (161, 162).

Yunnan has a suitable climate and has natural simulated BGS. Furthermore, this region has large bodies of water, which increases air humidity through evaporation and controls temperature by absorbing heat waves. The forest coverage rate in Yunnan exceeds 60%, and tropical rainforests, evergreen broad-leaved forests, and other vegetation release water through transpiration, maintaining air humidity, absorbing dust, and purifying the air. It is one of the zones with the highest worldwide climate diversity and species richness because of the proper proportion of BGS that act in concert to prevent the extreme heat in the low latitudes and to reduce the extreme cold at high elevations.

All regions around the world have recognized that BGS offers multiple benefits, including reducing floods and heat, and increasing biodiversity. The potential of blue-green infrastructure (BGI) to convert urban areas into sustainable and adaptable environments can be observed in several projects in Spain (163). Governments in regions affected by extreme climates can formulate targeted measures based on these research findings, such as increasing vegetation, maintaining green areas, optimizing green space forms, and paying attention to the ratio of BGS.

#### The prevention and control project for dengue fever

By 2023, there had been several dengue fever outbreaks in China’s Guangdong and Yunnan provinces. Incomplete statistics show that there were 114,853 instances in total, including cases that were imported and those that were transmitted locally (164). The prevalent dengue virus serotypes include DENV-1, DENV-2, DENV-3, and DENV-4. Furthermore, the diversity of virus types has also presented a great challenge for disease prevention and control (165). Despite the difficult path of dengue prevention and control, China still perseveres. The Ministry of Health and the Chinese Center for Disease Control and Prevention published the crucial document “Dengue Fever Monitoring Plan (Trial) and Dengue Fever Prevention Technical Guidelines” following the main “Healthy China” policy. The central and local governments jointly formulated the “National Dengue Fever Prevention and Control Guidelines.” During the outbreak of the epidemic, the state and various administrative departments demonstrated extraordinary leadership, immediately activating the emergency response mechanism and organizing multi-departmental coordination and cooperation. Multiple department levels demonstrated exceptional coordination capabilities: disease control departments at all levels improved case management and epidemic monitoring, ensuring that higher-level departments were able to rapidly grasp epidemic trends and make real-time adjustments to prevention and control strategies while also providing front-line staff with professional knowledge, guiding vector control, and educating the public about health issues. Furthermore, various medical and health departments responded quickly, assembling medical staff and medical supplies to support the epidemic front, as well as expert teams conducting disease research, creating guidelines for diagnosis and treatment, developing detection techniques, and developing therapeutic drugs. Moreover, other community administration departments carried out their assigned tasks, guaranteeing the smooth operation of food, hygiene, and transportation while supporting pertinent agencies with health education and epidemic surveillance. The inspection and quarantine departments collaborated to improve the screening and monitoring of imported and exported goods, as well as border ports, circulating goods, and mobile personnel for diseases. The financial department also ensured the support of epidemic prevention funds, and people from all walks of life and various fund organizations assisted, ensuring sufficient epidemic materials and funds (166).

China has achieved considerable progress in controlling dengue fever, as evidenced by the fact that, from 2005 to 2023, the country reported only 15 dengue fever-related deaths, with a fatality rate of fewer than 0.01 cases per 100,000 people (167). When the COVID-19 pandemic broke out in 2019, successful practical experience led to the “dynamic zero case” of the epidemic. The lessons that can be drawn include:

1. The government’s capable leadership: In addition to swiftly creating workable policies, plans, and action plans, the government was able to temporarily designate responsible individuals to oversee cross-departmental cooperation and successfully execute reward and punishment systems. These departments included public security, health, finance, and social welfare organizations.
2. Community organizations are strong forces for control and prevention: Community organizations can achieve a role transformation from passive acceptance of policies to active guidance of residents in prevention and control, with high efficiency in vector control and strong acceptability of health education, which can fully mobilize the enthusiasm of the masses to make up for the shortage of professional personnel.
3. Innovation in vector control: Based on traditional human trapping and light trapping, innovative measures such as chemical, biological, and environmental control are adopted to reduce mosquito density, interrupt virus transmission, including remote sensing and geographic space analysis (168).

Because of the significant experience in prevention and control, the epidemic has been effectively controlled. However, there are still many areas that require improvement and the adoption of advanced international technologies and experiences. Although the disease control department places a high priority on vector control management, and public awareness of mosquito prevention has grown, human health is still seriously threatened by mosquito-borne diseases. A recent study shows that using Wolbachia bacteria may inhibit the vectors or reduce their ability to spread various vector-borne viruses through solutions that induce conditional egg infertility and compete for survival with wild-type male mosquitoes (169). This novel modeling is crucial for the current global context of health issues.

#### Joint prevention and control project for flood-affected areas

In 2020, severe rainstorms occurred frequently in some areas of China, and a total of 37 floods were recorded (170). The regulation of water conservation facilities in urban areas is frequently restricted due to the dense population, flat terrain, and limited living environment in China’s vast territory, as well as the disparate economic development of different cities and significant geographical differences. To effectively reduce flood risks, it is crucial to develop a well-coordinated joint flood regulation model in cities, which is highly challenging. However, Chinese water experts are still actively analyzing the temporal and spatial clustering characteristics of China and formulating a storage and drainage joint regulation model suitable for urban areas in China’s plains.

To evaluate the flood storage and discharge management models of cities and to distribute the urban flood risk levels and storage-discharge capabilities of cities, recent studies presented a unique concept based on “jointly regulated urban agglomerations” by building multi-layer tree rules (171). The research results is as follows: First, risk assessment of jointly regulated urban agglomerations. A thorough evaluation of flood hazards is carried out, and the flood risk levels of cities are categorized based on the features of the regulating cities and their surrounding cities. The cities’ capacity to store and release floodwaters is fully understood. Second, analysis of flood coordination models. Through multi-layer tree rules, the storage-after-release (SD), release-after-storage (DS), and release-and-storage-at-the-same-time (DWS) models are allocated. For instance, a city with a large water storage capacity can use the SD model to reduce regional flood pressure while ensuring its own low risk. The DS model should be utilized to reduce flood pressure in towns with strong storage capacity, whereas the DWS model should be adopted for cities with weak storage capacity. Coastal cities can adopt the DS model to assist neighboring cities with high flood risks, while non-coastal cities can adopt the DWS model. Furthermore, the role of the urban agglomeration needs to be adjusted in real time according to the precipitation characteristics in the early, middle, and late stages of the rainstorm to better coordinate the flood diversion pressure. Third, an innovative management model. As a novel approach to rainwater management, green infrastructure (172) creates thorough and long-lasting evaluation metrics to address urban flooding and climate change.

For floods, different countries have proposed plans and policies that are suitable for them. For instance, Japan has constructed the world’s largest drainage channel to regulate floods (173). The urban area control policies of different countries vary, and to improve the flood control models, we should share and learn from one another.

#### Cross-regional joint ecological vulnerability response strategies

China-Myanmar border: Based on China’s “Malaria Elimination Action Plan (2010–2020)” and the actual needs of Myanmar, Health Poverty Action (HPA) was established in the border area of Yunnan Province. In the border region of Yunnan Province, a sustainable network for malaria prevention and control has been established thanks to the HPA’s tripartite model of multi-level management, local implementation, and cross-border cooperation (7). However, political unpredictability, poor staff subsidies, significant family responsibilities, and a lack of professional technical experience in multi-drug resistant malaria all have an impact on sustainability because HPA relies on outside funding; its work is extremely challenging. This model can serve as a valuable guide for future international cross-border health collaboration, but it must address problems, such as technical sustainability, staffing, and funding.

Environmental safety in the karst border region between Guangxi and Vietnam: The Ecological Security Pattern (ESP) solves the three main problems of ecological connectivity, human activity interference, and cultural adaptability in delicate ecological areas like karst by combining scientific quantification (such as the MCR model, circuit theory) with traditional ecological knowledge (TEK) (174). In cross-border ecological governance, the optimized “technology + tradition” model (such as the “three axes, two belts, six zones” framework in Guangxi) provides a reusable paradigm for the karst regions worldwide. ESP needs to seek a balance between “ecological rigidity protection” (such as the core area of karst) and “development elasticity demand” (such as border trade), and cross-border coordination and the modernization of TEK are the core levers to solve the problem. In the ecological security patterns study, the Minimum Cumulative Resistance (MCR) model and the Gravity model are the core methods for identifying and screening important ecological corridors. In addition to offering cross-regional coordination solutions, the MCR model also gives “physical connectivity” and “functional importance” based on the “physical connectivity” it provides. By combining the two, ecological corridors can be precisely screened, and vulnerable areas (like the Xiliu Ditch in the Yellow River Basin, China) can receive scientific support for ecological restoration (175).

## Recommendations

### Recommendations on the vulnerability of the health system

The urban public health system (U-PHS) vulnerability is a complex system that requires a comprehensive and holistic assessment. The recent study employed a combined assessment model that integrates the “human-machine-environment-process” four-dimensional framework and the “Bayesian best-worst method (B-BWM) + cloud model” to comprehensively evaluate factors that affect the vulnerability of U-PHS, and identified 18 factors. Among these, the most significant issues are the poor coordination and cooperation among personnel, insufficient information assurance, low public awareness, and low competence among employees of relevant departments and institutions (176). In the Southwestern region of China (especially Yunnan), infrastructure construction is challenging, the information network is not complete, the public health system is vulnerable, and health vulnerability is significant due to the underdeveloped economic level, complex geographic environment and climatic conditions, and a lack of public awareness and cognition. Yunnan’s flora and animal resources are abundant. According to an analysis of the public health system’s vulnerability issues, the frequent occurrence of wild mushroom and snake-bite poisoning incidents is attributed to the joint effect of the “human-machine-environment-management” four-dimensional factors. Currently, our other team members are thoroughly studying the two scientific research directions of snake bites and mushroom poisonings *via* epidemiological investigations, clinical treatment challenges analysis, networked construction to improve treatment levels and public cognition, and by establishing clinical prediction models, etc. We hope that future research successes can provide new scientific evidence for the challenges of ecosystem vulnerability in Yunnan.

### Recommendations on systemic vulnerable diseases

#### Zoonotic diseases

*Formulate corresponding laws and regulations:* Strengthen the reporting, control, and implementation of emergency response plans for zoonotic infectious diseases (177).

*Upgrade the infectious disease reporting system:* Various organizations, including hospitals, community health centers, the Ministry of Health, the Ministry of Forestry, and the Ministry of Agriculture, should work together and exchange information online to respond to infectious disease emergencies effectively and efficiently (178, 179).

*Implementation of special projects for major infectious diseases:* The effective integration of technology sharing platforms, professional academic alliances, typical demonstration areas, and high-quality forces can establish a multi-link network technology system and a strong support system for infectious disease prevention and control, as well as improve the emergency response capabilities for infectious diseases (180).

*Monitoring of abnormal diseases:* The monitoring of cross-border transmission of exotic diseases should be strengthened, and a cross-border animal disease control joint mechanism should be established between China and neighboring countries (181).

*Emphasize interdisciplinary and international cooperation:* An international early warning system should be established to timely monitor unknown infectious diseases in international public health (182).

*Health education:* Living habits should be changed, the living environment should be improved, and public awareness should be increased (183).

#### Common systemic vulnerable diseases

##### Strengthening the monitoring and early warning system

- *Climate Adaptation Warning:* The climate model predictions should be combined to forecast the risk of vector-borne diseases (such as the early warning system for African malaria).

##### Improving the healthcare system

- *Resource Fair Distribution:* Vaccine coverage should be improved in low-income countries through international assistance (Global Alliance for Vaccines and Immunization, GAVI).
- *Innovative Health Financing Mechanism:* Disease burden should be included in major policy evaluation systems by controlling the Health Impact Assessment (HIA) system. The primary barriers to action are a lack of resources and a shortage of workers. It is recommended that climate change be integrated into the development of primary health care, and that sustainable government should provide funding and resource support.
- *Primary Service:* The community medical worker model (such as the “Health Promotion Program” in Ethiopia) should be promoted.
- *Integrate Traditional Chinese and Western Medicine*: The prevention and treatment integration training bases should be established in regions with abundant traditional Chinese medicine resources (such as Gansu and Guangxi).

##### Cross-sectoral collaborative governance

- (a)*Environmental and Health Linkage*: Sanitation facilities should be improved (such as the “Clean India Movement” in India to reduce waterborne infectious diseases), environmental pollution during urbanization should be controlled, and meteorological and health data should be integrated to establish an early warning system.
- *Economic Policy Intervention*: The risk of infectious diseases should be reduced through poverty reduction (United Nations Sustainable Development Goal SDG1).

##### Public health education and behavioral intervention

- Health Education: The public should be educated on mosquito prevention measures, safe drinking water, and vaccination.
- Chronic Disease Control: High-risk behaviors should be changed through taxation (Mexican “Sugar-Sweetened Beverage Tax Law”) and legislation (anti-smoking policies).

##### Climate adaptation and resilience building

- Climate-Sensitive Disease Response: Adaptive technologies such as heat-resistant vaccines and anti-malaria nets should be developed.
- Disaster Emergency Response: A rapid response mechanism for cholera after floods (such as the case in Bangladesh) should be established.

##### Technological innovation and application

- *Digital Healthcare:* Artificial intelligence (AI) to predict disease outbreaks (such as modeling by Metabiota in the United States), mobile healthcare to cover remote areas, AI-assisted drug resistance prediction, microfluidic chip rapid detection, *etc.,* should be employed to improve diagnosis efficiency, establish an environment-genome monitoring database, and promote region-specific disease prevention and control plans.
- *Genetic Technology:* Genetic editing should be performed to control malaria transmission (such as Targe Malaria).
- *Application of Geographic Information Technology:* A Geographic Information System (GIS) should be adopted to draw disease risk maps, such as the malaria transmission risk spatial model established by the Chinese Center for Disease Control and Prevention.

##### Global cooperation

Chinese experience in chronic disease prevention and control should be promoted, international vaccine distribution and monitoring network governance measures should be established, and guidelines by the WHO and Global Infectious Disease Monitoring Network (GOARN) should be followed to share epidemic data, such as virus genome tracking observed during the COVID-19 pandemic.

### Future challenges

Ecosystems are changing due to climate change, and the effects of this change on human health are profound and far-reaching. Monitoring across time and space, using predictive models, studying the influencing mechanisms, formulating response strategies, implementing feasible plans, and achieving desired results are all lengthy and complex processes. Disease distribution occurs because of natural, economic, and social factors, and the policy response should integrate the concept of “One Health” (human-animal-environmental health integration) (184, 185), and achieve comprehensive management through multidisciplinary cooperation, international assistance, and localized deployment strategies. Therefore, establishing a more resilient public health system to meet the long-term challenges of climate change would require multifaceted and cross-scale structural adjustments.

## Footnotes

^1^ http://www.news.cn/20250715/e1cfa95744124efeb46966aa3bfbc66a/c.html

^2^ https://www.news.cn/politics/20250727/d2d840908baa489580f36723c8733f7b/c.html
